# DTI-LM: language model powered drug–target interaction prediction

**DOI:** 10.1093/bioinformatics/btae533

**Published:** 2024-09-02

**Authors:** Khandakar Tanvir Ahmed, Md Istiaq Ansari, Wei Zhang

**Affiliations:** Department of Computer Science, University of Central Florida, Orlando, FL 32816, United States; Genomics and Bioinformatics Cluster, University of Central Florida, Orlando, FL 32816, United States; Department of Computer Science, University of Central Florida, Orlando, FL 32816, United States; Genomics and Bioinformatics Cluster, University of Central Florida, Orlando, FL 32816, United States; Department of Computer Science, University of Central Florida, Orlando, FL 32816, United States; Genomics and Bioinformatics Cluster, University of Central Florida, Orlando, FL 32816, United States

## Abstract

**Motivation:**

The identification and understanding of drug–target interactions (DTIs) play a pivotal role in the drug discovery and development process. Sequence representations of drugs and proteins in computational model offer advantages such as their widespread availability, easier input quality control, and reduced computational resource requirements. These make them an efficient and accessible tools for various computational biology and drug discovery applications. Many sequence-based DTI prediction methods have been developed over the years. Despite the advancement in methodology, cold start DTI prediction involving unknown drug or protein remains a challenging task, particularly for sequence-based models. Introducing DTI-LM, a novel framework leveraging advanced pretrained language models, we harness their exceptional context-capturing abilities along with neighborhood information to predict DTIs. DTI-LM is specifically designed to rely solely on sequence representations for drugs and proteins, aiming to bridge the gap between warm start and cold start predictions.

**Results:**

Large-scale experiments on four datasets show that DTI-LM can achieve state-of-the-art performance on DTI predictions. Notably, it excels in overcoming the common challenges faced by sequence-based models in cold start predictions for proteins, yielding impressive results. The incorporation of neighborhood information through a graph attention network further enhances prediction accuracy. Nevertheless, a disparity persists between cold start predictions for proteins and drugs. A detailed examination of DTI-LM reveals that language models exhibit contrasting capabilities in capturing similarities between drugs and proteins.

**Availability and implementation:**

Source code is available at: https://github.com/compbiolabucf/DTI-LM.

## 1 Introduction

In the relentless pursuit of novel therapeutic agents, the intricate interplay between drugs and their biological targets has become the focal point of modern pharmaceutical research. The concept of drug–target interaction (DTI) constitutes the cornerstone of contemporary drug discovery and development, providing a fundamental framework for understanding the mechanistic foundations of pharmacological interventions. Amid the ever-evolving challenges posed by drug resistance and adverse drug reactions, the exploration of DTI not only expedites the identification of novel drug candidates but also augments our capacity to repurpose existing compounds for diverse therapeutic applications. Experimental assays have proven to be the gold standard for DTI identification (Zheng *et al.* 2020). However, research indicates that the expenses associated with the development of new drugs vary between $314 million and $2.8 billion, while the duration of clinical development typically spans between 8.2 and 10.0 years (Wouters *et al.* 2020, Brown *et al.* 2021). These substantial investments in time and resources have made DTI prediction an indispensable tool to aid the initial stages of drug discovery by expediting the identification of potential drug–target interactions, thereby streamlining the process of lead compound selection and, consequently, experimental validation.

Numerous studies have demonstrated the utility of computational approaches, including machine learning algorithms, network-based methods, and molecular docking simulations for DTI prediction. In recent times, the advancement of DTI prediction has been notably accelerated, primarily attributed to the extensive accumulation and accessibility of biomedical datasets. This surge is further propelled by the remarkable progress of deep learning techniques, which have showcased exceptional success across diverse realms of scientific research and asserted themselves as the predominant method for DTI prediction. Several advanced deep learning-based frameworks for DTI prediction have emerged, utilizing diverse sets of data as input. These frameworks can be broadly categorized into knowledge graph-based methods (Luo *et al.* 2017, Thafar *et al.* 2020, Ye *et al.* 2021, Zhang *et al.* 2023), 3D structure-based approaches (Wallach *et al.* 2015, Ragoza *et al.* 2017, Stepniewska-Dziubinska *et al.* 2018, Khodabandeh Yalabadi *et al.* 2024, Bian *et al.* 2024, Wang *et al.* 2024), 2D pairwise distance map-based techniques (Zheng *et al.* 2020, Li *et al.* 2022), and 1D sequence-based methods (Wen *et al.* 2017, Öztürk *et al.* 2018, Chen *et al.* 2020, Huang *et al.* 2021). Heterogeneous knowledge graph (KG)-based methods have demonstrated success in various scenarios of DTI prediction, including warm start, cold start for drugs, and cold start for proteins. Cold start predictions involving unknown drugs or proteins are particularly challenging as limited or no information about that drug or protein is available during model training. Despite this challenge, KG-based models leverage semantic relationships with other entities (such as shared pathways, biological processes, or functional annotations) and diverse data sources, enabling them to achieve competitive performance in cold start predictions. However, it’s crucial to note that KG-based methods demand large amounts of heterogeneous datasets and substantial computational resources to achieve state-of-the-art results. Their performance is also contingent on the completeness of the knowledge graph. Structure and sequence-based methods generally tend to perform worse for cold start predictions if the cold start protein or drug has no structural or sequential homologs with known interactions in training. Moreover, obtaining high-quality structural data for all proteins of interest can be challenging and time-consuming and requires significant computational resources. On the contrary, 1D sequences, such as amino acid sequences for proteins and Simplified Molecular Input Line Entry System (SMILES) for drugs, represent the most readily available form of input data and require less computation due to their simplified representation. Ensuring the quality of data is also more straightforward compared to knowledge graphs and structural information. Therefore, addressing the limitations associated with cold start problems using 1D sequences holds the potential to accurately predict interactions for a broader spectrum of drugs and proteins compared to other methods.

The adoption of pretrained language models (LMs) has emerged as a transformative tool across a spectrum of research domains. BERT (Bidirectional Encoder Representations from Transformers) (Devlin *et al.* 2018) brought about a paradigm shift in natural language processing tasks, and its impact extended to other domains such as ESM, ProtBert, and ProteinBERT (Elnaggar *et al.* 2021, Brandes *et al.* 2022, Lin *et al.* 2023) for protein feature extraction. Similarly, in drug-related contexts, models like ChemBERTa, ChemGPT, and MoLFormer (Chithrananda *et al.* 2020, Ross *et al.* 2022, Frey *et al.* 2023) have played a crucial role in extracting drug features. These pretrained models have found applications and validation in previous DTI prediction studies, wherein embeddings are generated utilizing LMs (Kalakoti *et al.* 2022, Kang *et al.* 2022, Nguyen *et al.* 2022). These embeddings generated by LMs are independent, meaning no neighborhood information is considered during their generation. While such approaches have proven effective, recent studies, including those utilizing KG-based frameworks, have demonstrated the efficacy of neighborhood-based embedding generation for DTI prediction (Wan *et al.* 2019). Incorporating neighborhood information into language model-based embeddings has the potential to yield improved representations for both drugs and proteins. Moreover, previous language model-based DTI prediction studies (Kalakoti *et al.* 2022, Nguyen *et al.* 2022) lack a comprehensive comparison with other methods, focusing only on the comparison among the language model variants.

In this study, we introduce a novel framework, DTI-LM, designed for predicting drug–target interactions by leveraging language models to generate encodings from protein amino acid and drug SMILES sequences. Going beyond traditional approaches, we enhance the encoding process by introducing graph attention networks (GAT). These networks enrich the representations of proteins and drugs with neighborhood information, thereby contributing to more nuanced and context-aware DTI predictions. Our experimental findings substantiate the effectiveness of the proposed DTI-LM framework, demonstrating superior performance compared to existing state-of-the-art DTI prediction models while utilizing fewer data and computational resources. Furthermore, we design our study to investigate the current limitations associated with language model-based DTI prediction. We shed light on the difference in performance between cold start for proteins and drugs and probe into the bottleneck for cold start for drugs prediction. This exploration allows us to gain insights into the challenges and boundaries that currently exist in protein and drug language models, providing a foundation for potential future enhancements and refinements in language model-based drug–target interaction prediction.

## 2 Materials and methods

In this section, we first introduce the mathematical notations used in this study, followed by the proposed framework, DTI-LM. The framework can take protein amino acid sequences and drug SMILES sequences as inputs in language models, followed by graph attention networks and a multi-layer perceptron (MLP) to predict DTIs. We then discuss the baselines used in this study to illustrate the improvements offered by our model.

### 2.1 Overview of the framework

**Figure 1.**
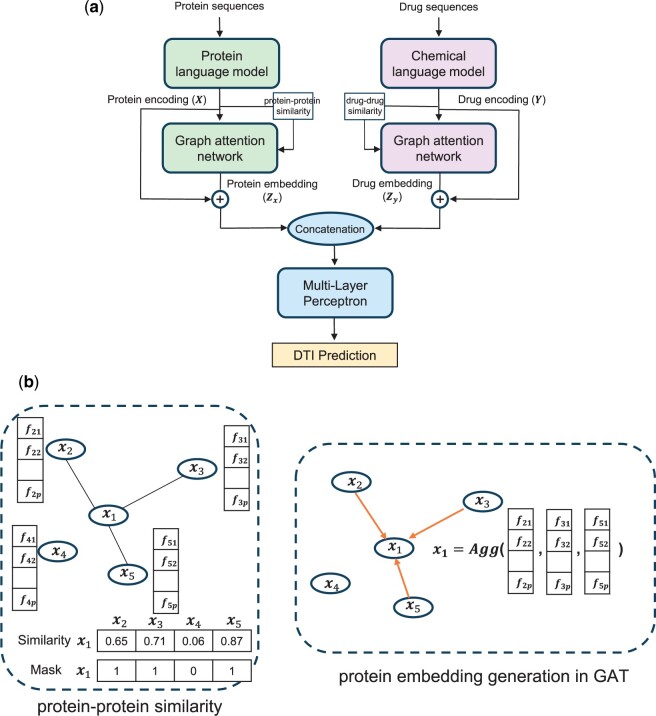
(a) Overall framework of DTI-LM. In the framework, protein and drug sequences are fed into their respective language models. Next, the generated encoding and their similarity matrix are used in a graph attention network to generate protein and drug embeddings. The embeddings are then concatenated and passed into a multi-layer perceptron to predict DTI. (b) GAT embedding generation. It illustrates the process for protein embedding generation using a GAT from LLM encoding of proteins. Neighbors with high similarity are aggregated to update the target node embedding. We follow the same procedure for drug embedding generation.

In the context of language model-based DTI prediction frameworks, the protein embeddings produced by protein language models are inherently distinct for each protein sequence, just as the drug embeddings generated by chemical language models remain independent for different drug sequences (Kalakoti *et al.* 2022). Although similar proteins or drugs should generate similar embeddings, enhancements to these embeddings can be achieved by explicitly defining a neighborhood based on similarities or interactions between drugs or proteins. Conversely, in GAT-based DTI prediction frameworks, various encoding methods such as integer encoding, Word2Vec, position-specific scoring matrix, or biological property-based encoding are utilized to prepare the protein sequences. For drug sequences, encodings like molecular fingerprint, molecular graph, and Word2Vec are used as input for the GAT model (Wang *et al.* 2021, Zhang *et al.* 2021, Jiang *et al.* 2022, Cheng *et al.* 2022, Wang *et al.* 2023). As a step toward an integrated approach, we propose combining both strategies by encoding the protein and drug sequences using language models and subsequently generating the final representations through the GAT model. Figure 1 illustrates the overall workflow of DTI-LM.

The notations used to define the proposed model are summarized in Table 1. Let $X=[x_{1},x_{2},\ldots ,x_{m}]$ represent the *p*-dimensional encodings for *m* proteins generated by the protein language model from protein sequences represented by amino acids, where $x_{i}$ denotes the *i*th protein. Similarly, $Y=[y_{1},y_{2},\ldots ,y_{n}]$ represents the *q*-dimensional encodings for *n* drugs generated from drug SMILES sequences. $Z_{x}$ and $Z_{y}$ are GAT protein and drug embeddings, respectively, where *k*, *l*, and *h* represent the protein embedding size, drug embedding size, and the number of heads in the GAT. The proposed framework is designed for binary prediction of the drug–target interaction matrix, denoted by I. For the remainder of the manuscript, outputs from the LMs are designated as encodings, and outputs from the GATs are designated as embeddings to easily differentiate between them.

**Table 1.** Notations used in DTI-LM.

Name	Definition
*p*, *q*, *m*, *n*, *k*, *l*, *h*	Protein encoding size, drug encoding size, number of proteins, number of drugs, protein GAT embedding size, drug GAT embedding size, number of heads respectively
$X\in R^{p\times m}$	Protein sequence encoding generated by ESM-2
$Y\in R^{q\times n}$	Drug SMILES encoding generated by ChemBERTa
$S_{x}\in R^{m\times m}$	Protein–protein adjacency matrix
$S_{y}\in R^{n\times n}$	Drug–drug adjacency matrix
$Z_{x}\in R^{kh\times m}$	Protein embeddings generated by GAT
$Z_{y}\in R^{lh\times n}$	Drug embeddings generated by GAT
$I\in R^{m\times n}$	Drug–target interaction matrix

#### 2.1.1 Protein encoding

We use ESM-2 (Lin *et al.* 2023), a 33-layer, 650-million-parameters model with an output dimension of 1280 for encoding protein sequences. It is an advanced deep-learning model specifically designed to capture the complex evolutionary patterns and structural features embedded within protein sequences. The model is trained on the UniRef50 dataset, which is part of the UniProt Knowledgebase (The UniProt Consortium 2022), a centralized repository for protein sequences and functional information. The dataset is constructed through the clustering of UniRef90 seed sequences, ensuring that each cluster comprises sequences with a minimum of 50% sequence identity to, and 80% overlap with, the longest sequence in the cluster and consists of 11 862 245 clusters (Suzek *et al.* 2015). By encoding protein sequences using ESM-2, we can harness the model’s capacity to capture long-range dependencies and subtle sequence motifs, thereby facilitating more accurate predictions of protein properties, functions, and interactions. ESM-2 was chosen over other protein language models such as AlphaFold2, RoseTTAFold, OmegaFold, ProtBert, etc. (Baek *et al.* 2021, Elnaggar *et al.* 2021, Jumper *et al.* 2021, Wu *et al.* 2022) due to its faster runtime and high-quality embedding generation. The computational cost of multiple sequence alignment (MSA) powered models (AlphaFold2, RoseTTAFold) can be one to two orders of magnitude higher than ESM-2 (Lin *et al.* 2023) while offering negligible advantage in DTI prediction accuracy (Kalakoti *et al.* 2022).

#### 2.1.2 Drug encoding

For drug SMILES sequence encoding, we choose a prominent chemical language model, ChemBERTa (Chithrananda *et al.* 2020), a 6-attention layer, 84-million-parameters model with an output dimension of 768. It was trained on 10 million SMILES sequences from the PubChem database (Kim *et al.* 2023). ChemBERTa integrates the powerful language understanding capabilities of BERT with domain-specific knowledge from the chemical and pharmaceutical realms. By encoding drug SMILES sequences, ChemBERTa enables the extraction of rich semantic representations, capturing intricate molecular structures, functional groups, and chemical properties embedded within the SMILES notations. With its capacity to comprehend complex chemical structures and their relationships, ChemBERTa serves as a valuable tool for drug discovery. In this study, we implemented our model using the Hugging Face library (HuggingFace 2023), a widely recognized and extensively utilized platform for natural language processing and deep learning research.

#### 2.1.3 Drug–target interaction prediction

Protein and drug encodings, given by X and Y, respectively, are fed into two GATs to derive embeddings by integrating neighborhood information. To define the neighborhood of a protein, an m×m Pearson correlation matrix $S_{x}$ is first calculated. This correlation-based similarity matrix is then converted into a binary adjacency matrix using a threshold where high correlation scores above that threshold are assigned value of 1 while low scores below that threshold are assigned value of 0. The binarized adjacency matrix will be later used to mask the attention coefficients of the model. Whether to keep self-connections in the adjacency matrix and the thresholds used for binarization are set as hyperparameters in the framework and tuned for the best performance. All hyper parameters of the model and details of the adjacency matrix calculation process are presented in the Supplementary Document. A similar process is applied to obtain the drug neighborhood $S_{y}$. The model can accommodate other neighborhood definitions such as the protein-protein interaction network (PPI) and drug-drug interaction network (DDI). Once we have the adjacency matrices, we can generate the embeddings for X and Y. For protein embedding, the attention directed to $x_{i}$ from its neighbor $x_{j}$ can be computed as follows:


(1) $$c_{ij}=a[Wx_{i}||Wx_{j}],$$

where $W\in R^{k\times p}$ and $a\in R^{1\times 2k}$ represent the learnable weight parameters of a single head. Here, *k* denotes the embedding size of the GAT, and ‖ denotes the concatenation operation. Subsequently, the calculated attention values undergo a *LeakyReLU* activation function. To incorporate the structural information of the network, the attention values are modified by applying a mask using the adjacency matrix. Specifically, only the attention values corresponding to connected nodes in the adjacency matrix $S_{x}$ are retained, while all other values are set to zero. The attention coefficient for a neighbor $x_{j}$ is then calculated using the *Softmax* function as follows:


(2) $$\alpha_{ij}=\frac{\;\text{exp}(\mathrm{LeakyReLU}(c_{ij}))}{\sum_{r\in N_{i}}\;\text{exp}\;(\mathrm{LeakyReLU}(c_{ir}))},$$

where $N_{i}$ represents the neighborhood of the $i^{th}$ protein. The embedding of $x_{i}$ is calculated as:


(3) $${x^{\prime }}_{i}=\sigma (\sum_{j\in N_{i}}\alpha_{ij}Wx_{j}),$$

where σ is a nonlinear activation function. We use multi-head attention mechanism to capture complex relationships and enhance the expressiveness of the learned representations. For *h* number of heads, each with its separate attention mechanism, the final embedding of the sample is obtained by concatenating the output of the heads. Therefore, the final embedding of the *i*th protein is given by:


(4) $$z_{i}=|{|}_{h=1}^{h}\sigma (\sum_{j\in N_{i}}\alpha_{ij}^{h}W^{h}x_{j}).$$

We obtain the embeddings for all *m* proteins as $Z_{x}\in R^{kh\times m}$ and follow the same procedure to obtain the embeddings for *n* drugs as $Z_{y}\in R^{lh\times n}$, where *l* is the embedding size for drugs from a single head. We design the GAT model to have the same embedding size as LM encoding, i.e. kh=p and lh=q. For simplicity, we show same number of heads *h* for drugs and proteins which can be different in implementation of DTI-LM. The number of heads and number of layers in the networks used for generating protein and drug embeddings are set as hyperparameters in the model.

Finally, the protein embedding $Z_{x}$ and the encoding from the language model X are added together to obtain the final protein representations. Similarly, the drug embedding $Z_{y}$ and the encoding from the language model Y are added together to obtain the final drug representations. These representations are concatenated and fed into a multilayer perceptron (MLP) to predict the corresponding interactions, as given by:


(5) $$\tilde{I}=\mathrm{MLP}([Z_{x}+\beta X]||[Z_{y}+\gamma Y]).$$

β and γ are hyperparameters that control the contribution of the residual connection. The model is trained with binary cross-entropy loss, calculated as:


(6) $$L=-\frac{1}{mn}\sum_{i=0}^{mn}[I_{i}\cdot \;\text{log}\;\sigma (\tilde{I_{i}})+(1-I_{i})\cdot \;\text{log}(\sigma (1-\tilde{I_{i}}))],$$

where σ represents the *Sigmoid* function.

### 2.2 Baselines models

We use several baselines to compare the performance of our proposed model, DTI-LM. DeepDTA (Öztürk *et al.* 2018), DeepDTI (Wen *et al.* 2017), and TransDTI (Kalakoti *et al.* 2022) are end-to-end models that take protein and drug sequences as input, similar to DTI-LM. DeepDTA and DeepDTI use convolutional neural networks and deep belief networks, respectively, to process the protein and drug sequences. TransDTI, on the other hand, uses language models for protein and drug sequences with an MLP on top of the outputs from the language models. MolTrans (Huang *et al.* 2021) uses transformers with sequence data and FragXsiteDTI (Khodabandeh Yalabadi *et al.* 2024) uses GCN and transformers with 3D structures for DTI prediction. In addition, DTI-LM is compared against heterogeneous data-driven models such as DTiGEMS+ (Thafar *et al.* 2020), DTINet (Luo *et al.* 2017), KGE_NFM (Ye *et al.* 2021), and TriModel (Mohamed *et al.* 2021) that require more data modalities to train than DTI-LM. Although DTI-LM uses protein-protein and drug-drug similarity matrices, we can generate these matrices from the language model encoding without any external information.

## 3 Experiments

### 3.1 Dataset

The proposed framework is evaluated on four datasets: DrugBank (Law *et al.* 2014), BindingDB (Liu *et al.* 2007), Yamanishi_08 (Yamanishi *et al.* 2008), and Luo’s dataset (Luo *et al.* 2017). The DrugBank and BindingDB datasets contain only protein and drug sequences; therefore, they were primarily utilized for comparing sequence-based methods. In contrast, the Yamanishi_08 and Luo’s datasets include heterogeneous knowledge graphs (KG) alongside protein and drug sequences, making them suitable for comparing both sequence-based and heterogeneous data-driven methods. The Yamanish_08 network encompasses 25 487 nodes and 95 579 edges, whereas Luo’s dataset network consists of 12 015 nodes and 1 895 445 edges. Statistics of the datasets can be found in Table 2.

**Table 2.** Data statistics.

Dataset	Proteins	Drugs	KG	Interactions
DrugBank	2203	1603	No	6041
BindingDB	879	9144	No	4040
Yamanishi_08	722	791	Yes	3448
Luo’s	1129	708	Yes	1526

### 3.2 Running DTI-LM

First, the DrugBank and BindingDB datasets are split into training, validation, and test sets, with ratios of 0.79, 0.01, and 0.20, respectively. This splitting process adheres to three specific conditions: warm start (the same drugs and proteins being allowed in both training and test sets), cold start for drugs (drugs in training and test sets are exclusive), and cold start for proteins (proteins in training and test sets are exclusive). The Yamanishi_08 and Luo’s datasets are obtained from the source mentioned in Ye *et al.* (2021), and the same training and test splits as utilized in that study are used to generate our results. While sequence-based models, including DTI-LM, are exclusively trained on the sequences, heterogeneous data-driven models incorporate the use of KG as well. Therefore, heterogeneous data-driven models are not compared on DrugBank and BindingDB datasets. DrugBank, Yamanishi_08, and Luo’s datasets provide binary interaction details that were used in our classification framework to train a binary classifier to predict interaction or no interaction for a pair of drug and protein. In contrast, BindingDB provides binding affinity (Kd) data, which is converted into a binary format using a threshold to align with the classification framework. The threshold is chosen to maintain a comparable DTI density as other datasets. The hyperparameters of the framework are fine-tuned using Ray Tune (Liaw *et al.* 2018), and comprehensive information regarding the selection of hyperparameters can be found in the Supplementary Document (Supplementary Table S4). All predictions are run 10 times with different splittings, with the mean area under the Receiver Operating Characteristic curve (AUROC) and the area under the Precision-Recall curve (AUPRC) reported in the respective tables. These experiments are repeated with two variations in the ratios of positive and negative samples in the datasets: balanced data has a 1:1 ratio, whereas unbalanced data has a 1:10 ratio between positive and negative drug–target pairs or all samples if the ratio is less than 1:10.

DTI-LM is thoroughly evaluated through various experiments. Firstly, we compare the performance of DTI prediction with cutting-edge baselines, highlighting the improvements introduced by our model. Subsequently, we conduct an in-depth analysis of DTI-LM to examine its benefits and drawbacks, specifically focusing on the use of the language model-based encoding for DTI prediction.

### 3.3 Prediction results

We designed two DTI prediction scenarios to illustrate the ability of DTI-LM. Firstly, we conducted a comparative analysis of our model against other sequence-based models using DrugBank and BindingDB datasets, demonstrating the enhanced predictive capabilities of our approach relying solely on sequence data. We repeated the experiments with all three types of splitting, each with balanced and unbalanced datasets. Secondly, we pitted our model against heterogeneous data-driven models using Yamanishi_08 and Luo’s datasets, highlighting our competitive performance despite utilizing only a fraction of the input data. Not only is protein and drug sequence data more readily available, but it can also significantly reduce the computational complexity of a model compared to heterogeneous data-driven models. In Tables 3–6, the first row associated with each splitting strategy represents the AUROC, while the second row depicts the AUPRC.

**Table 3.** The classification performance on DrugBank dataset.^a^

		DTI-LM	TransDTI	DeepDTA	DeepDTI
Balanced	Warm start	**0.951**	0.934	0.889	0.916
		**0.953**	0.935	0.882	0.914
	Cold start for drug	**0.902**	0.877	0.874	0.859
		**0.899**	0.889	0.871	0.868
	Cold start for protein	**0.923**	0.916	0.855	0.838
		**0.935**	0.920	0.825	0.850
Unbalanced	Warm start	**0.960**	0.952	0.907	0.947
		**0.863**	0.858	0.623	0.773
	Cold start for drug	**0.890**	0.876	0.765	0.860
		**0.674**	0.651	0.441	0.582
	Cold start for protein	**0.938**	0.916	0.737	0.871
		**0.821**	0.789	0.441	0.614

a Average AUROC and AUPRC scores of drug–target prediction for warm start, cold start for drug, and cold start for protein data splitting. The most significant results are bolded.

**Table 4.** The classification performance on BindingDB dataset.^a^

		DTI-LM	TransDTI	DeepDTA	DeepDTI
Balanced	Warm start	**0.939**	0.926	0.868	0.923
		**0.934**	0.918	0.729	0.910
	Cold start for drug	**0.872**	0.870	0.754	0.863
		0.879	0.878	0.699	**0.886**
	Cold start for protein	**0.812**	0.809	0.697	0.757
		**0.787**	0.779	0.572	0.767
Unbalanced	Warm start	**0.945**	0.941	0.820	0.935
		**0.839**	0.834	0.577	0.813
	Cold start for drug	0.895	0.872	0.851	**0.896**
		**0.744**	0.708	0.637	0.743
	Cold start for protein	0.831	0.818	**0.869**	0.761
		0.463	0.456	**0.568**	0.366

a Average AUROC and AUPRC scores of drug–target prediction for warm start, cold start for drug, and cold start for protein data splitting. The most significant results are bolded.

**Table 5.** The classification performance on Yamanishi_08 dataset.^a^

		Sequences-based					Structure-based		Heterogeneous data-driven		
		DTI-LM	TransDTI	DeepDTI	MPNN_CNN	MolTrans	FragXsiteDTI (Predicted)	FragXsiteDTI (Mixed)	DTiGEMS+	TriModel	KGE_NFM
Balanced	Warm start	**0.974**	0.969	0.865	0.834	0.885	0.838	0.700	0.964	0.951	0.968
		**0.966**	0.961	0.820	0.788	0.837	0.870	0.741	0.957	0.946	0.961
Unbalanced	Warm start	0.984	0.984	0.982	0.974	0.926	0.905	0.848	0.976	**0.985**	0.983
		**0.930**	0.927	0.917	0.874	0.658	0.708	0.451	0.874	0.886	0.902
	Cold start for drug	0.785	0.762	0.628	0.629	0.733	0.742	0.814	0.745	0.817	**0.853**
		0.451	0.442	0.191	0.194	0.288	0.371	**0.741**	0.518	0.503	0.521
	Cold start for protein	0.911	0.902	0.497	0.502	0.568	0.483	0.417	0.674	0.829	**0.921**
		**0.739**	0.729	0.099	0.098	0.103	0.097	0.061	0.443	0.483	0.679

a Average AUROC and AUPRC scores of drug–target prediction for warm start, cold start for drug, and cold start for protein data splitting. The most significant results are bolded. DeepDTI, MPNN_CNN, DTiGEMS+, TriModel, and KGE_NFM results are directly reproduced from Ye *et al.* (2021).

**Table 6.** The classification performance on Luo’s dataset.^a^

		Sequences-based					Structure-based		Heterogeneous data-driven	
		DTI-LM	TransDTI	DeepDTI	MPNN_CNN	MolTrans	FragXsiteDTI (Predicted)	FragXsiteDTI (Mixed)	DTINet	KGE_NFM
Balanced	Warm start	**0.944**	0.938	0.859	0.830	0.906	0.838	0.844	0.940	0.903
		**0.948**	0.939	0.840	0.805	0.915	0.870	0.864	0.941	0.898
Unbalanced	Warm start	**0.971**	**0.971**	0.952	0.929	0.914	0.905	0.917	0.944	0.962
		**0.906**	0.902	0.793	0.705	0.694	0.708	0.751	0.817	0.855
	Cold start for drug	0.760	0.742	0.662	0.806	0.658	0.742	0.731	0.853	**0.881**
		0.393	0.383	0.225	0.462	0.241	0.371	0.376	**0.592**	0.555
	cold start for protein	**0.832**	0.823	0.487	0.431	0.529	0.477	0.425	0.778	0.813
		**0.595**	0.589	0.092	0.078	0.110	0.114	0.097	0.388	0.444

a Average AUROC and AUPRC scores of drug–target prediction for warm start, cold start for drug, and cold start for protein data splitting. The most significant results are bolded. DeepDTI, MPNN_CNN, DTINet, and KGE_NFM results are directly reproduced from Ye *et al.* (2021).

The results presented in Tables 3 and 4 showcase the average classification results of the sequence-based model applied to the DrugBank and BindingDB datasets, respectively. They highlight that our model outperformed the baseline models in the majority of cases. Notably, under the warm start scenario, our model consistently demonstrated superior performance compared to all the baselines across both datasets. The most substantial performance enhancement was observed in the case of cold start for protein splitting despite doing worse than DeepDTA in unbalanced BindingDB dataset. Across different splitting scenarios, our model exhibited an average improvement in AUROC of 3.57% and AUPRC of 8.33% for warm start, 3.84% and 6.13% for cold start for drug, and 5.57% and 8.93% for cold start for protein predictions, respectively. AUROC scores are better in unbalanced splittings due to higher volume of training data. AUPRC scores are unsurprisingly lower for unbalanced splittings as there are far less positive interactions compared to negative interactions that makes positive interaction predictions more challenging. We also find that DeepDTA is more unstable compared to other models with a large gap of performance between balanced and unbalanced splitting. It works better for balanced data in DrugBank while doing better for unbalanced data in BindingDB.

Next, Tables 5 and 6 report the average classification results for sequence-based, structure-based and heterogeneous data-driven models on Yamanishi_08 and Luo’s datasets. Using the same publicly available data splits as Ye *et al.* (2021) enables a direct comparison of our results with those reported in that paper. FragXsiteDTI suffers from a common shortcoming of structure-based methods, the unavailability of protein structures. Therefore, we ran FragXsiteDTI twice, once with all predicted protein structures (Predicted). Then we repeat the experiment with experimental protein structure if available, and predicted protein structure otherwise (Mixed). ESMFold (Lin *et al.* 2023) was used to predict the protein structures that uses similar underlying technique as ESM-2. As observed in the Tables, heterogeneous data-driven baselines DTiGEMS+, DTINet, TriModel, and KGE_NFM consistently outperform sequence-based baselines DeepDTI, MolTrans, and MPNN_CNN and structure-based model FragXsiteDTI across various scenarios, with a notable performance gap for cold start for drug and cold start for protein splittings. Despite being a sequence-based model, DTI-LM not only outperforms other sequence-based baselines but also surpasses and heterogeneous data-driven models for warm start and cold start for protein prediction. For cold start for drug splitting, while we outperform other sequence-based and structure-based baselines in most cases, except FragXsiteDTI on the Yamanishi_08 dataset and MPNN_CNN on Luo’s dataset, we still lag behind state-of-the-art heterogeneous data-driven models. This underscores the findings from Tables 3 and 4 that DTI-LM is more effective for cold start for protein splitting than cold start for drug splitting. To gain a deeper understanding of the factors contributing to the superior performance of our model in the context of cold start for protein as opposed to cold start for drug, we conducted an investigation detailed in Section 3.5.

### 3.4 Transition from cold start to warm start

**Figure 2.**
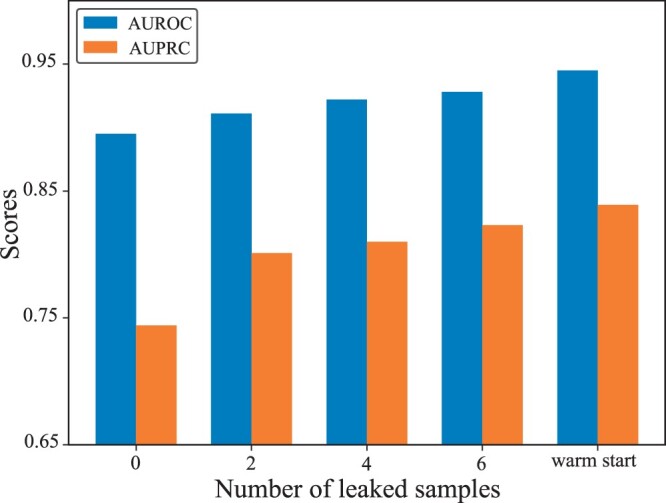
Effect of leaked samples. AUROC and AUPRC scores after 2, 4, and 6 samples leaked into training of cold start for drug prediction.

Given the limitations in cold start for drug splitting, we investigated the transition between a cold start and warm start prediction to determine the minimum information needed for the transition. For each drug in the test set, we sent a number of samples (drug–target pair) to the training set and tracked how the prediction performance changes with the inclusion of additional information. All predictions with leaked data are also computed 10 times similar to previous results. Figure 2 illustrates the results for the DrugBank dataset, where we leaked two, four, and six samples from each drug in the test set to the training set but kept at least one sample for those drugs in the test set. AUPRC has a larger gap between warm start and cold start scenario compared to AUROC. The figure shows that, AUPRC jumps significantly with inclusion of just two samples on average for each test drug that is comparable to warm start predictions. Both AUROC and AUPRC keep gradually increasing as we leak more samples.

### 3.5 Language model encoding analysis

In this section, we examine the current strengths and weaknesses of language model-based DTI prediction. As observed in the results reported above, DTI-LM performs better in warm start and cold start for protein predictions but lags behind in cold starts for drug predictions. In contrast, other 1D sequence-based methods struggle with both cold starts for protein and cold starts for drug predictions. For cold start predictions, performance depends on how much the model can learn about an unknown drug or protein from the known drugs or proteins in the training data. The results suggest that DTI-LM effectively learned representations for unknown proteins, given the high AUROC and AUPRC values in cold starts for protein prediction. However, it fails to replicate a similar level of learning for unknown drugs. If the representations are significantly different in the training and test sets for a pair of drugs that share similar interactions, this difference can explain the poor performance in cold starts for drug prediction. Therefore, we compute the similarity of drugs and proteins using their respective SMILES and amino acid sequences, as well as the encoding generated by language models, to inspect the efficiency of the language models in finding similar drugs and proteins.

Table 7 shows the similarity of drugs and proteins in the benchmark datasets. For drug similarity using SMILES sequences, we utilize the RDKit library (RDKit 2023) to measure Tanimoto similarity on Morgan fingerprints. Clustal Omega (Sievers *et al.* 2011) is used to determine amino acid sequence similarity for proteins. On the other hand, for language model encoding similarities, we calculate the Pearson correlation for each pair of drugs or proteins separately, based on the representations generated by the language models. This process generates two m×m protein-protein similarity matrices and two n×n drug-drug similarity matrices. The mean similarity for all drug/protein pairs is reported in Table 7. As shown, neither drug nor protein sequences exhibit significant similarity. It’s important to note that sequence-level drug and protein similarity is not directly comparable. However, both similarity metrics have a range of 0–1, with 1 indicating the highest similarity. The lack of significant similarity is evident. In contrast, the language model encodings are highly similar across all datasets, particularly in the case of protein encoding. This underscores the greater ability of the protein language model (ESM-2) to capture protein similarity even when amino acid sequences are not very similar. However, it remains a possibility that ESM-2 generates all protein encodings similarly, regardless of the actual similarity between them, which may impede DTI prediction. Therefore, we conduct another experiment to investigate whether similar drugs or proteins in the encoding domain also share similar interactions. We measure how many drug-protein interactions of a given drug (or protein) are supported by the majority of its neighboring drugs (or proteins). Neighbors are defined as the top N similar drugs (proteins) to a drug (protein) using raw sequence or encoding-based similarity matrices. In this experiment, we set N = 5, and a protein (drug) interaction of a given drug (protein) must be shared by at least three of its neighboring drugs (proteins).

**Table 7.** Sequence and encoding similarity.^a^

	Raw sequences		LM encoding	
Dataset	drug	protein	drug	Protein
DrugBank	0.101	0.072	0.644	0.853
BindingDB	0.117	0.090	0.574	0.859
Yamansihi_08	0.104	0.089	0.554	0.853
Luo’s dataset	0.097	0.078	0.488	0.845

a Similarity is measured based on the raw sequences and language model encodings representing drugs and proteins.

Table 8 presents the average percentage of interactions supported by the majority (three or more) of neighbors for a drug or protein. We use both raw sequence-based similarities and encoding-based similarities to construct the neighborhood. From the table, we can see that drugs receive a higher percentage of support from neighbors compared to proteins when neighbors are selected based on raw sequence-based similarity. However, the average percentage of support for drugs decreases across all datasets when neighbors are selected based on language model encoding. This suggests that encoding similarity in drugs is less meaningful, as similar drugs may exhibit drastically different interactions.

**Table 8.** Top five neighbor support.^a^

	Raw sequences		LM encoding	
Dataset	Drug	Protein	Drug	Protein
DrugBank	25.1%	0.0%	14.3%	30.7%
Yamansihi_08	14.1%	21.5%	6.5%	44.0%
Luo’s dataset	30.9%	0.0%	24.5%	26.4%

a Average percentage of interactions shared by majority of the neighbors.

Table 8 also illustrates the noteworthy increase in average percentage of support for proteins using similarity matrix generated from language model encoding compared to raw sequence. For example, 44% of all drug-protein interactions from proteins in Yamanishi_08 dataset are also shared by at least three of their respective neighbor proteins. The presence of a strong neighborhood led us to use GAT to incorporate this vital information in the DTI prediction and our implementation of GAT successfully improves the prediction performance over TransDTI. In light of these findings, we can see why DTI-LM demonstrates substantial improvements in cold start for proteins predictions but faces challenges in the case of drugs. Existing chemical language models may struggle to capture the complex interwoven information in the SMILES sequences as efficiently as ESM-2 does for protein sequence.

## 4 Discussion

In our comprehensive experiments, DTI-LM shows great prediction results, especially for warm start and cold start for proteins scenarios. It successfully overcomes the traditional challenges faced by sequence-based models for cold start for protein prediction. However, it falls short of achieving a comparable level of performance for cold start for drugs, despite improvements over the existing sequence-based models. We delved deeply into analyzing the reasons for the discrepancies between cold start for protein and drug predictions. This exploration would help us understand whether the limitations in cold start for drug prediction stem from the constraints of current chemical language models or our proposed architecture. Our experiments, detailed in Section 3.5, show that the ESM-2 is very effective in finding similar proteins that also share similar drug interactions based solely on amino acid sequences. In contrast, ChemBERTa lacks the same level of proficiency for drugs. We also explored the performance of newer, larger models such as ChemGPT (Frey *et al.* 2023) and observed similar outcomes.

The experiment outlined in Section 3.5 is not conclusive; instead, it gives us a general idea about the performance of the protein and chemical language models. A few crucial aspects of the experiment are discussed below.

- In Table 7, we present the Pearson correlation, which ignores the nonlinear relationship that can be captured by the subsequent GAT and MLP we use for the prediction.
- The average neighbor support, as shown in Table 8, paints an important but incomplete picture. The training process involves contributions from samples beyond the top five neighbors, impacting results irrespective of the quality of these neighbors.
- Finding support for protein interaction and drug interaction may also pose varying levels of difficulty due to the different numbers of drugs and proteins in each dataset. For instance, datasets like DrugBank and Luo’s exhibit a lower number of proteins than drugs, i.e. proteins have fewer options to choose from to find an interaction than drugs. Therefore, the probability of proteins sharing similar interactions will be higher than drugs sharing similar interactions. This circumstance can make it comparatively easier to find neighbor proteins with similar drug interactions than neighbor drugs with similar protein interactions. However, Yamanishi_08 has more drugs than proteins (as indicated in Table 2) while having the largest difference between support for proteins and drugs, as seen in Table 8. Therefore, the difference cannot be completely explained by the number of proteins or drugs.
- It is possible that drugs with similar sequences inherently do not share similar interactions. This makes finding drugs with similar interactions based solely on sequences more challenging. However, we use the support for drug interactions based on raw sequences as a baseline (Table 8) and expect the language models to capture more complex similarities. We observe that ESM-2 aligns with this expectation, showing an improved percentage of support in LM encoding compared to raw sequences. On the other hand, ChemBERTa fails to meet the expectation and demonstrates lower support for LM encoding compared to raw sequences. This could be interpreted as similar drug LM encodings being further away from sharing similar interactions than similar SMILES sequences.

The domain of pre-trained language models is improving at an unprecedented level, giving us hope for stronger and more advanced chemical language models in the future. This progress is expected to address cold start for drugs issues more effectively, as ESM-2 has done for cold start for protein predictions.

Based on the higher percentage of support for drugs using raw sequences in Table 8, we utilized a raw sequence-based similarity matrix in drug GAT for DTI prediction and found worse results (results are not shown in the manuscript). This can be attributed to the fact that similar SMILES sequences can have different LM encodings; thus, the raw sequence-based neighborhood will be less meaningful for LM encoding. These limitations might be prevalent in all language model-based DTI prediction frameworks that use drug sequence data. In addition, to further investigate the quality of LM encodings, we conducted zero-shot DTI predictions relying only on the neighborhood information. The experimental details are reported in the Supplementary Document (Supplementary Tables S1–S3). The results show that zero-shot prediction consistently outperforms sequence-based baselines and occasionally outperforms heterogeneous data-driven models, indicating the useful information contained within drug-drug or protein-protein similarity matrix based neighborhoods.

## 5 Conclusion

We propose DTI-LM, a language model-based DTI prediction framework that incorporates neighborhood information for predictions. Our goal is to achieve state-of-the-art results in various prediction scenarios and to test the limits of existing protein and chemical language models for these tasks. DTI-LM outperformed the baselines for warm start and cold start for protein predictions. We also tracked back on the weak performance of DTI-LM for cold start for drug predictions and identified the chemical language model as a limiting factor. Recent notable advancements in natural language processing may pave the way for the development of improved protein and chemical language models to address the cold start problem more efficiently. Nevertheless, DTI-LM currently excels in cold start for protein predictions, a crucial aspect for personalized medicine where tailoring treatment to individual patients’ protein variants is essential.

## Supplementary Material

btae533_Supplementary_Data

## Data Availability

The code and datasets of DTI-LM are available at https://github.com/compbiolabucf/DTI-LM/.

## References

[btae533-B1] Baek M , DiMaio F, Anishchenko I et al Accurate prediction of protein structures and interactions using a three-track neural network. Science 2021;373:871–6. 34282049 10.1126/science.abj8754 PMC7612213

[btae533-B2] Bian J , Lu H, Dong G et al Hierarchical multimodal self-attention-based graph neural network for DTI prediction. Brief Bioinform 2024;25:bbae293. 10.1093/bib/bbae293 PMC11200190 38920341

[btae533-B3] Brandes N , Ofer D, Peleg Y et al ProteinBERT: a universal deep-learning model of protein sequence and function. Bioinformatics 2022;38:2102–10. 35020807 10.1093/bioinformatics/btac020 PMC9386727

[btae533-B4] Brown DG , Wobst HJ, Kapoor A et al Clinical development times for innovative drugs. Nat Rev Drug Discov 2021;21:793–4. 10.1038/d41573-021-00190-9 PMC9869766 34759309

[btae533-B5] Chen L , Tan X, Wang D et al TransformerCPI: improving compound–protein interaction prediction by sequence-based deep learning with self-attention mechanism and label reversal experiments. Bioinformatics 2020;36:4406–14. 32428219 10.1093/bioinformatics/btaa524

[btae533-B6] Cheng Z , Yan C, Wu F-X et al Drug–target interaction prediction using multi-head self-attention and graph attention network. IEEE/ACM Trans Comput Biol Bioinform 2022;19:2208–18. 33956632 10.1109/TCBB.2021.3077905

[btae533-B7] Chithrananda S , Grand G, Ramsundar B. ChemBERTa: large-scale self-supervised pretraining for molecular property prediction. arXiv, arXiv:2010.09885, 2020, preprint: not peer reviewed.

[btae533-B8] Devlin J , Chang M-W, Lee K et al Bert: Pre-training of deep bidirectional transformers for language understanding. arXiv, arXiv:1810.04805, 2018, preprint: not peer reviewed.

[btae533-B9] Elnaggar A , Heinzinger M, Dallago C et al ProtTrans: toward understanding the language of life through self-supervised learning. IEEE Trans Pattern Anal Mach Intell 2021;44:7112–27. 10.1109/TPAMI.2021.3095381 34232869

[btae533-B10] Frey NC , Soklaski R, Axelrod S et al Neural scaling of deep chemical models. Nat Mach Intell 2023;5:1297–305.

[btae533-B11] Huang K , Xiao C, Glass LM et al MolTrans: molecular interaction transformer for drug–target interaction prediction. Bioinformatics 2021;37:830–6. 33070179 10.1093/bioinformatics/btaa880 PMC8098026

[btae533-B12] HuggingFace. Hugging face. https://huggingface.co/ (26 October 2023, date last accessed).

[btae533-B13] Jiang L , Sun J, Wang Y et al Identifying drug–target interactions via heterogeneous graph attention networks combined with cross-modal similarities. Brief Bioinform 2022;23:bbac016. 35224614 10.1093/bib/bbac016

[btae533-B14] Jumper J , Evans R, Pritzel A et al Highly accurate protein structure prediction with AlphaFold. Nature 2021;596:583–9. 34265844 10.1038/s41586-021-03819-2 PMC8371605

[btae533-B15] Kalakoti Y , Yadav S, Sundar D et al TransDTI: transformer-based language models for estimating DTIs and building a drug recommendation workflow. ACS Omega 2022;7:2706–17. 35097268 10.1021/acsomega.1c05203 PMC8792915

[btae533-B16] Kang H , Goo S, Lee H et al Fine-tuning of Bert model to accurately predict drug–target interactions. Pharmaceutics 2022;14:1710. 36015336 10.3390/pharmaceutics14081710 PMC9414546

[btae533-B17] Kim S , Chen J, Cheng T et al PubChem 2023 update. Nucleic Acids Res 2023;51:D1373–80. 36305812 10.1093/nar/gkac956 PMC9825602

[btae533-B18] Law V , Knox C, Djoumbou Y et al DrugBank 4.0: shedding new light on drug metabolism. Nucleic Acids Res 2014;42:D1091–7. 24203711 10.1093/nar/gkt1068 PMC3965102

[btae533-B19] Li F , Zhang Z, Guan J et al Effective drug–target interaction prediction with mutual interaction neural network. Bioinformatics 2022;38:3582–9. 35652721 10.1093/bioinformatics/btac377 PMC9272808

[btae533-B20] Liaw R , Liang E, Nishihara R et al Tune: a research platform for distributed model selection and training. arXiv, arXiv:1807.05118, 2018, preprint: not peer reviewed.

[btae533-B21] Lin Z , Akin H, Rao R et al Evolutionary-scale prediction of atomic-level protein structure with a language model. Science 2023;379:1123–30. 36927031 10.1126/science.ade2574

[btae533-B22] Liu T , Lin Y, Wen X et al BindingDB: a web-accessible database of experimentally determined protein–ligand binding affinities. Nucleic Acids Res 2007;35:D198–201. 17145705 10.1093/nar/gkl999 PMC1751547

[btae533-B23] Luo Y , Zhao X, Zhou J et al A network integration approach for drug–target interaction prediction and computational drug repositioning from heterogeneous information. Nat Commun 2017;8:573. 28924171 10.1038/s41467-017-00680-8 PMC5603535

[btae533-B24] Mohamed SK , Nounu A, Nováček V et al Biological applications of knowledge graph embedding models. Brief Bioinform 2021;22:1679–93. 32065227 10.1093/bib/bbaa012

[btae533-B25] Nguyen TM , Nguyen T, Tran T et al Mitigating cold-start problems in drug–target affinity prediction with interaction knowledge transferring. Brief Bioinform 2022;23:bbac269. 35788823 10.1093/bib/bbac269 PMC9353967

[btae533-B26] Öztürk H , Özgür A, Ozkirimli E et al DeepDTA: deep drug–target binding affinity prediction. Bioinformatics 2018;34:i821–9. 30423097 10.1093/bioinformatics/bty593 PMC6129291

[btae533-B27] Ragoza M , Hochuli J, Idrobo E et al Protein–ligand scoring with convolutional neural networks. J Chem Inf Model 2017;57:942–57. 28368587 10.1021/acs.jcim.6b00740 PMC5479431

[btae533-B28] RDKit. RDKit: Open-source cheminformatics. https://www.rdkit.org (26 October 2023, date last accessed).

[btae533-B29] Ross J , Belgodere B, Chenthamarakshan V et al Large-scale chemical language representations capture molecular structure and properties. Nat Mach Intell 2022;4:1256–64.

[btae533-B30] Sievers F , Wilm A, Dineen D et al Fast, scalable generation of high-quality protein multiple sequence alignments using Clustal Omega. Mol Syst Biol 2011;7:539. 21988835 10.1038/msb.2011.75 PMC3261699

[btae533-B31] Stepniewska-Dziubinska MM , Zielenkiewicz P, Siedlecki P et al Development and evaluation of a deep learning model for protein–ligand binding affinity prediction. Bioinformatics 2018;34:3666–74. 29757353 10.1093/bioinformatics/bty374 PMC6198856

[btae533-B32] Suzek BE , Wang Y, Huang H et al; UniProt Consortium. UniRef clusters: a comprehensive and scalable alternative for improving sequence similarity searches. Bioinformatics 2015;31:926–32. 25398609 10.1093/bioinformatics/btu739 PMC4375400

[btae533-B33] Thafar MA , Olayan RS, Ashoor H et al DTiGEMS+: drug–target interaction prediction using graph embedding, graph mining, and similarity-based techniques. J Cheminform 2020;12:44–17. 33431036 10.1186/s13321-020-00447-2 PMC7325230

[btae533-B34] The UniProt Consortium. UniProt: The Universal Protein Knowledgebase in 2023. Nucleic Acids Res 2022;51:D523–31. 10.1093/nar/gkac1052 PMC9825514 36408920

[btae533-B35] Wallach I , Dzamba M, Heifets A. AtomNet: a deep convolutional neural network for bioactivity prediction in structure-based drug discovery. arXiv, arXiv:1510.02855, 2015, preprint: not peer reviewed.

[btae533-B36] Wan F , Hong L, Xiao A et al NeoDTI: neural integration of neighbor information from a heterogeneous network for discovering new drug–target interactions. Bioinformatics 2019;35:104–11. 30561548 10.1093/bioinformatics/bty543

[btae533-B37] Wang H , Zhou G, Liu S et al Drug–target interaction prediction with graph attention networks. arXiv, arXiv:2107.06099, 2021, preprint: not peer reviewed.

[btae533-B38] Wang J , Xiao Y, Shang X et al Predicting drug–target binding affinity with cross-scale graph contrastive learning. Brief Bioinform 2024;25:bbad516. 10.1093/bib/bbad516 PMC10788681 38221904

[btae533-B39] Wang K , Hu J, Zhang X et al Identifying drug–target interactions through a combined graph attention mechanism and self-attention sequence embedding model. In: *International Conference on Intelligent Computing*. Singapore: Springer Nature Singapore, 2023, 246–57.

[btae533-B40] Wen M , Zhang Z, Niu S et al Deep-learning-based drug–target interaction prediction. J Proteome Res 2017;16:1401–9. 28264154 10.1021/acs.jproteome.6b00618

[btae533-B41] Wouters OJ , McKee M, Luyten J et al Estimated research and development investment needed to bring a new medicine to market, 2009-2018. JAMA 2020;323:844–53. 32125404 10.1001/jama.2020.1166 PMC7054832

[btae533-B42] Wu R , Ding F, Wang R et al High-resolution de novo structure prediction from primary sequence. bioRxiv, 10.1101/2022.07.21.500999, 2022, preprint: not peer reviewed.

[btae533-B43] Khodabandeh Yalabadi A , Yazdani-Jahromi M, Yousefi N et al FragXsiteDTI: revealing responsible segments in drug–target interaction with transformer-driven interpretation. In: *International Conference on Research in Computational Molecular Biology*. Cham: Springer Nature Switzerland, 2024, 68–85.

[btae533-B44] Yamanishi Y , Araki M, Gutteridge A et al Prediction of drug–target interaction networks from the integration of chemical and genomic spaces. Bioinformatics 2008;24:i232–40. 18586719 10.1093/bioinformatics/btn162 PMC2718640

[btae533-B45] Ye Q , Hsieh C-Y, Yang Z et al A unified drug–target interaction prediction framework based on knowledge graph and recommendation system. Nat Commun 2021;12:6775. 34811351 10.1038/s41467-021-27137-3 PMC8635420

[btae533-B46] Zhang R , Wang Z, Wang X et al MHTAN-DTI: metapath-based hierarchical transformer and attention network for drug–target interaction prediction. Brief Bioinform 2023;24:bbad079. 36892155 10.1093/bib/bbad079

[btae533-B47] Zhang S , Jiang M, Wang S et al SAG-DTA: prediction of drug–target affinity using self-attention graph network. Int J Mol Sci 2021;22:8993. 34445696 10.3390/ijms22168993 PMC8396496

[btae533-B48] Zheng S , Li Y, Chen S et al Predicting drug–protein interaction using quasi-visual question answering system. Nat Mach Intell 2020;2:134–40.

