# Supplementary material for: DTI-LM: language model powered drug–target interaction prediction
Source: Bioinformatics. 2024 Sep 2;40(9):btae533. doi: 10.1093/bioinformatics/btae533 (PMC11520403; doi:10.1093/bioinformatics/btae533)
Supplement: btae533_Supplementary_Data [file btae533_supplementary_data.pdf]

DTI-LM: Language Model Powered Drug-Target Interaction Prediction

Zero-shot prediction

In zero-shot learning, our objective is to predict test samples that belong to classes unseen by the model during its training phase. In the specific context of binary DTI prediction, the model faces the challenge of being trained exclusively on either all positive or all negative drug-target pairs. An alternative approach involves bypassing the training phase that requires labels and directly inferring DTIs based on representations of drugs and targets. In the case of DTI-LM, we leverage language model encodings generated from pretrained models, eliminating the need for labeled data while capturing neighborhood information, as illustrated in Table 8. Therefore, we design a zero-shot experiment to assess predictive ability without explicitly training a model for cold start drug/protein prediction.

Experimental design

For cold start for protein prediction, let $\boldsymbol{y}_{tr}$ and $\boldsymbol{y}_{te}$ represent the train and test set respectively. $\boldsymbol{y}_{tr}$ is a tuple of $[\boldsymbol{P}_{tr}, \boldsymbol{D}_{tr}, \boldsymbol{I}_{tr}]$ where $\boldsymbol{P}_{tr}$ are the proteins, $\boldsymbol{D}_{tr}$ are drugs, and $\boldsymbol{I}_{tr} \in \{0,1\}^{|\boldsymbol{P}_{tr}|\times|\boldsymbol{D}_{tr}|}$ are the drug-protein interactions in the train set respectively. Similarly, $\boldsymbol{y}_{te} = [\boldsymbol{P}_{te}, \boldsymbol{D}_{te}, \boldsymbol{I}_{te}]$ contains the proteins, drugs and their interactions in the test set. Considering a cold start for protein prediction, $\boldsymbol{P}_{tr}$ and $\boldsymbol{P}_{te}$ are not allowed to have overlapped proteins but $\boldsymbol{D}_{tr}$ and $\boldsymbol{D}_{te}$ can have overlapped drugs. First, we generate protein and drug sequence encoding $\boldsymbol{X}$ and $\boldsymbol{Y}$ from the language models similar to DTI-LM. Pair-wise Pearson correlation matrix $\boldsymbol{S}_x$ is calculated from protein encoding $\boldsymbol{X}$. For a protein p in $\boldsymbol{P}_{te}$, we find the top $|\boldsymbol{N}|$ similar proteins denoted by $\boldsymbol{P}_{\boldsymbol{N}}$ from $\boldsymbol{P}_{tr}$ where $\boldsymbol{N}$ denotes the set of top neighbors. In this experiment we used $|\boldsymbol{N}| = 80$. We calculate the weighted labels $\boldsymbol{I}_w \in \{0,1\}^{|\boldsymbol{P}_{\boldsymbol{N}}|\times|\boldsymbol{D}_{tr}|}$ for all drug-target pairs associated with $\boldsymbol{P}_{\boldsymbol{N}}$ by multiplying the labels $\boldsymbol{I}_{tr}[\boldsymbol{P}_{\boldsymbol{N}}]$ with correlations of its associated proteins. The resultant weighted labels are aggregated for each drug and normalized to a range of $0-1$ to obtain the final likelihood of DTI. This predicted label is used with ground truth to measure AUROC and AUPRC scores. We follow similar procedure for cold start for drug prediction. The process for cold start for protein prediction is given in Algorithm 1.

The results of the zero-shot prediction are reported in Tables S1, S2, and S3. As an extension of Table 8 in the manuscript, we only use the datasets used in that table. All results worse than the zero-shot prediction are

Algorithm 1: Zero-shot prediction

Data: $\boldsymbol{X}$,$\boldsymbol{y}_{tr}$,$\boldsymbol{y}_{te}$,$\boldsymbol{N}$
Result: $\boldsymbol{I}_{pred}$
$\boldsymbol{X}$ is protein encoding, $\boldsymbol{y}_{tr}$ is train set, $\boldsymbol{y}_{te}$ is test set,
$\boldsymbol{N}$ is the set of neighbors, $\boldsymbol{I}_{pred}$ is predicted labels.
$\boldsymbol{S}_x \leftarrow$ calculateCorrelation($\boldsymbol{X}$);
$\boldsymbol{S}_x[\,:, \boldsymbol{P}_{te}] \leftarrow 0$; //columns in $\boldsymbol{S}_x$ corresponding to $\boldsymbol{P}_{te}$ are made 0 to ensure only proteins from $\boldsymbol{P}_{tr}$ can be selected as top neighbors
Initialize empty vector $\boldsymbol{I}_{pred}$;
for p *in* $\boldsymbol{P}_{te}$ **do**
  $\boldsymbol{s}_x \in [0,1]^{1\times|\boldsymbol{P}_{tr}|} \leftarrow \boldsymbol{S}_x[p]$;
  Find the top neighbors $\boldsymbol{P}_{\boldsymbol{N}} \leftarrow \boldsymbol{s}_x$;
  $\boldsymbol{I}_w \leftarrow \boldsymbol{I}_{tr}[\boldsymbol{P}_{\boldsymbol{N}}] \odot \boldsymbol{s}_x[\boldsymbol{P}_{\boldsymbol{N}}]^T$; //$T$ represents transpose operation
  $\boldsymbol{I}_p \leftarrow Normalize($ RowSum $(\boldsymbol{I}_w))$;
  Append $\boldsymbol{I}_p$ to $\boldsymbol{I}_{pred}$;

made italic. As seen in the tables, sequence-based baselines DeepDTA, DeepDTI, MolTrans, and MPNN_CNN as well as structure-based baseline FragXsiteDTI perform worse than the zero-shot prediction in most cases across DrugBank, Yamanishi_08, and Luo's dataset. DTI-LM, TransDTI, and the heterogeneous data-driven models perform better than the zero-shot prediction, except for DTiGEMS+ in the Yamanishi_08 dataset. It is noteworthy that these results do not necessarily correlate with Table 8 in the manuscript. Table 8 was calculated for each drug or protein in a leave-one-out approach, whereas Tables S1, S2, and S3 use the same train-test split as the other models in the tables, predicting interactions only for test drugs/proteins using train drugs/proteins. The zero-shot results emphasize the generalizability of the language model encodings as well as their usability when task-specific or dataset-specific labels are limited.

		DTI-LM	TransDTI	DeepDTA	DeepDTI	Zero-shot
balanced	cold start	0.902	0.877	0.874	0.859	0.805
	for drug	0.899	0.889	0.871	0.868	0.809
	cold start	0.923	0.916	*0.855*	*0.838*	0.870
	for protein	0.935	0.920	*0.825*	*0.850*	0.871
unbalanced	cold start	0.890	0.876	*0.765*	0.860	0.810
	for drug	0.674	0.651	*0.441*	*0.582*	0.602
	cold start	0.938	0.916	*0.737*	0.871	0.868
	for protein	0.821	0.789	*0.441*	*0.614*	0.706

Table S1: **The classification performance on DrugBank dataset.** Average AUROC and AUPRC scores of drug-target prediction for random, cold start for drug, and cold start for protein data splitting.

		sequences-based						Structure-based		heterogeneous data-driven		
		DTI-LM	TransDTI	DeepDTI	MPNN_CNN	MolTrans	Zero-shot	FragXsiteDTI (Predicted)	FragXsiteDTI (Mixed)	DTiGEMS+	TriModel	KGE_NFM
unbalanced	cold start	0.785	0.762	*0.628*	*0.629*	*0.733*	0.759	*0.742*	0.814	*0.745*	0.817	0.853
	for drug	0.451	0.442	*0.191*	*0.194*	*0.288*	0.395	*0.371*	0.741	0.518	0.503	0.521
	cold start	0.911	0.902	*0.497*	*0.502*	*0.568*	0.750	*0.483*	*0.417*	*0.674*	0.829	0.921
	for protein	0.739	0.729	*0.099*	*0.098*	*0.103*	0.474	*0.097*	*0.061*	*0.443*	0.483	0.679

Table S2: **The classification performance on Yamanishi_08 dataset.** Average AUROC and AUPRC scores of drug-target prediction for random, cold start for drug, and cold start for protein data splitting. DeepDTI, MPNN_CNN, DTiGEMS+, TriModel, and KGE_NFM results are directly reproduced from [1].

		sequences-based						Structure-based		heterogeneous data-driven	
		DTI-LM	TransDTI	DeepDTI	MPNN_CNN	MolTrans	Zero-shot	FragXsiteDTI (Predicted)	FragXsiteDTI (Mixed)	DTINet	KGE_NFM
unbalanced	cold start	0.760	0.742	*0.662*	0.806	*0.658*	0.719	0.742	0.731	0.853	0.881
	for drug	0.393	0.383	*0.225*	0.462	*0.241*	0.361	0.371	0.376	0.592	0.555
	cold start	0.832	0.823	*0.487*	*0.431*	*0.529*	0.544	*0.477*	*0.425*	0.778	0.813
	for protein	0.595	0.589	*0.092*	*0.078*	*0.110*	0.178	*0.114*	*0.097*	0.388	0.444

Table S3: **The classification performance on Luo's dataset.** Average AUROC and AUPRC scores of drug-target prediction for random, cold start for drug, and cold start for protein data splitting. DeepDTI, MPNN_CNN, DTINet, and KGE_NFM results are directly reproduced from [1].

Hyperparameter tuning

Table S4 lists all hyperparameters that were tuned for the best performance of DTI-LM. We used Ray Tune with 'Optuna' search algorithm and 'ASHA' scheduler for 100 trials to optimize the hyperparameters.

Table S4: Hyperparameters

hyperparameter	Values
learning rate	0.00001 - 0.01
batch size	[128, 256, 512, 768, 1024, 1280]
dropout	[0.2, 0.3, 0.4, 0.5]
MLP layers	[[512,256], [256,128], [1024,512,256], [512,256,128], [256,128,64], [1024,512,256,128], [512,256,128,64], [256,128,64,32], [2048,1024,256,128]]
activation	[ReLU, Sigmoid]
optimizer	[Adam, SGD]
weight decay	0.00001 - 0.001
β	[0.1, 1, 5, 10]
γ	[0.1, 1, 5, 10]
Drug GAT:	
heads	[2, 4, 8]
dropout	[0.1, 0.2, 0.3]
add self loops	[True, False]
num layers	[1, 2, 3, 4]
threshold for $\boldsymbol{S}_y$	0.1 - 0.9
Prot GAT:	
heads	[2, 4, 8]
dropout	[0.1, 0.2, 0.3]
add self loops	[True, False]
num layers	[1, 2, 3, 4]
threshold for $\boldsymbol{S}_x$	0.1 - 0.9

Adjacency matrix calculation

We calculate protein and drug similarity matrices for the graph attention networks in DTI-LM. The performance of the proposed model depends on the criteria and integrity of the constructed similarity matrices. We use Pearson's correlation to measure pairwise distance between proteins or drugs. Let $\boldsymbol{S}$ be the similarity matrix for proteins that is defined as:

$$\boldsymbol{S}_{ij} = \begin{cases} 1, & \text{if } Correlation(\boldsymbol{x}_i, \boldsymbol{x}_j) \geq T \\ 0, & \text{otherwise} \end{cases}$$

where $\boldsymbol{S}_{ij}$ is the connection between i^{th} and j^{th} proteins. $Correlation(\boldsymbol{x}_i, \boldsymbol{x}_j)$ calculates the Pearson correlation between the language model encoding of ith and j^{th} proteins and checks it against a threshold T. T is a hyper parameter of the model as mentioned in Table S4. This is repeated for all m protein to build up an $m \times m$ adjacency matrix and repeated for all n drugs to construct an $n \times n$ adjacency matrix.

References

[1] Qing Ye, Chang-Yu Hsieh, Ziyi Yang, Yu Kang, Jiming Chen, Dongsheng Cao, Shibo He, and Tingjun Hou. A unified drug–target interaction prediction framework based on knowledge graph and recommendation system. *Nature communications*, 12(1):6775, 2021.
